# Utilizing process mining in quality management: A case study in radiation oncology

**DOI:** 10.1371/journal.pdig.0000647

**Published:** 2025-05-15

**Authors:** Mohammad Bakhtiari

**Affiliations:** WellSpan Radiation Oncology, Chambersburg, Pennsylvania, United States of America; Instituto Politécnico Nacional Escuela Superior de Medicina: Instituto Politecnico Nacional Escuela Superior de Medicina, MEXICO

## Abstract

Radiation oncology is known for its complexity, inherent risks, and sheer volume of data. Adopting a process-oriented management approach and systemic thinking is essential for ensuring safety, efficiency, and the highest quality of care. Process mining offers a data-centric method for analyzing and improving clinical workflows to ensure optimal patient outcomes. This study utilizes process mining techniques along with a quality management system to analyze event logs obtained from an electronic medical record system. Conformance checking and process improvement methodologies were utilized to detect inefficiencies and bottlenecks. Examining the treatment planning process through process mining revealed two principal bottlenecks—OAR contouring and physics chart checks. This led to specific interventions that markedly decreased the time to complete treatment planning processes. Additionally, applying organizational mining methods provided valuable information on how resources are utilized and how teams collaborate within the organization. Process mining is a useful tool for improving efficiency, quality, and decision-making in radiation oncology. By transitioning from traditional management to a data-driven leadership approach, radiation oncology departments can optimize workflows, enhance patient care, and adapt to the evolving demands of modern healthcare.

## Author summary

In this study, we explored how process mining—a method commonly used in business—can improve quality and safety in healthcare. We focused on radiation therapy, a complex treatment that involves many professionals, steps, and technologies. These steps are recorded in electronic systems but are often difficult to visualize or fully understand. By applying process mining, we were able to map how care actually flows, uncover delays and inefficiencies, and identify patterns that may contribute to errors or extra work. Our aim was to make this process more transparent and easier to improve. Turning everyday data into clear, evidence-based insights can help healthcare teams work more safely and effectively. While this work focused on radiation oncology, the same approach could be used in other areas of healthcare to support better, more reliable patient care.

## 1 Introduction

Radiation oncology is a rapidly advancing field characterized by increasing complexity that often exceeds human cognitive abilities to manage effectively [1]. Over the past several decades, the volume of healthcare data has exponentially grown [2], necessitating systematic and data-driven approaches to quality management and decision-making. Professional organizations such as the American Association of Physicists in Medicine (AAPM) Task Group 100 [3] and frameworks from the American Society for Radiation Oncology (ASTRO) [4], along with other major healthcare guidelines [5,6], have emphasized the importance of process-oriented and data-driven methodologies as critical components of system thinking. According to system thinking safety and quality in healthcare are properties of well-designed systems [5]. System thinking requires a proper infrastructure for health information flow and data-driven decision-making [5].

In this context, we introduce the application of process mining [7] to radiation oncology as a potential tool to improve quality, safety, and leadership efficiency. Process mining has already been utilized in various healthcare sectors [8,9]. The methodology seeks to discover, evaluate, and enhance actual processes by deriving insights from event data [10]. Event data, tasks performed within a process accompanied by timestamps, are vastly available in information systems like Electronic Medical Records (EMR) or Treatment Planning Systems (TPS) [9,10]. However, according to the Data, Information, Knowledge, and Wisdom (DIKW) framework [11], large amount of raw data is valuable but meaningless unless purposely structured. Event data can be compiled into event logs, transforming them into informative assets, the initial stage of process mining [12]. Process mining techniques provide deep insights into workflows, identifying bottlenecks, deviations, and performance issues. This enables employees to anticipate and diagnose compliance problems, supporting automating or eliminating repetitive or outdated tasks as it is one of the focuses of big data and Artificial Intelligence applications [13–18].

The applications of process mining in radiation oncology are manifold, ranging from retrospective analyses, such as identifying the root causes of bottlenecks, to prospective predictions, such as estimating the remaining processing time of treatments or recommending actions to reduce safety issues. Process mapping and process mining, while related, have distinct differences. Process mapping involves manually creating diagrams representing the flow of activities within a process, often based on expert knowledge and observations [3]. Manual process mining is crucial to risk assessment, especially for new procedures or implementing new technologies. In contrast, process mining automatically discovers, monitors, and improves actual processes, offering an objective and dynamic view by analyzing event logs to uncover hidden patterns, identify inefficiencies, and suggest areas for improvement from existing processes with greater accuracy. Process mining bridges the gap between data science and process science [12]. Process science is a broad field that includes studying and applying methods to model, analyze, and improve processes. It encompasses various tools and techniques, including Petri nets, Directly-Follows Graphs (DFG), and Business Process Model and Notation (BPMN), which represent and analyze workflows. Section 1.2 will briefly present some of the process science tools.

**Fig 1:**
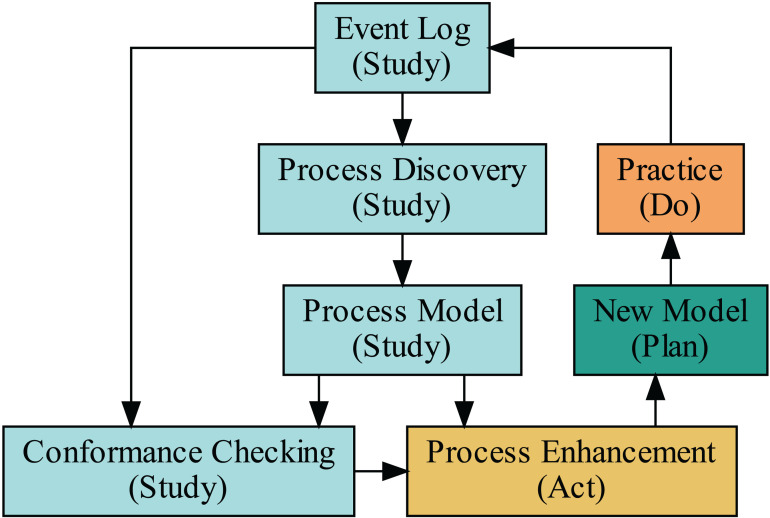
Framing process mining into the Plan-Do-Study-Act (PDSA) cycle can enhance the quality management system by providing detailed insights into existing processes. 1. Plan: Use process mining to develop a process model. 2. Do: Implement the planned process while continuously monitoring the process using process mining and other quality management tools to ensure adherence to the new model. 3. Study: Analyze the data collected during the implementation phase to evaluate the effectiveness of the changes. Process mining can highlight deviations and areas that need further improvement. 4. Act: Based on the analysis, make necessary adjustments to the process. Use the insights to refine the plan and prepare for the next cycle.

Process mining methodology includes three fundamental types: process discovery (section 1.3), conformance checking (section 1.4), and process enhancement (section 1.5). Additionally, we classify organizational mining (section 1.5.4) as a sub-branch of process enhancement. The process mining framework can be integrated into the Plan-Do-Study-Act (PDSA) cycle for continuous quality improvement [19–21], as illustrated in Fig 1. The PDSA cycle is a component of the Total Quality Management (TQM) approach, emphasizing iterative testing and learning [22]. One might also employ the Define-Measure-Analyze-Improve-Control (DMAIC) methodology within Six Sigma, which is known for its data-driven and structured approach [23]. Nevertheless, in this context, we opt for PDSA because of its simplicity in an introductory study.

**Fig 2:**
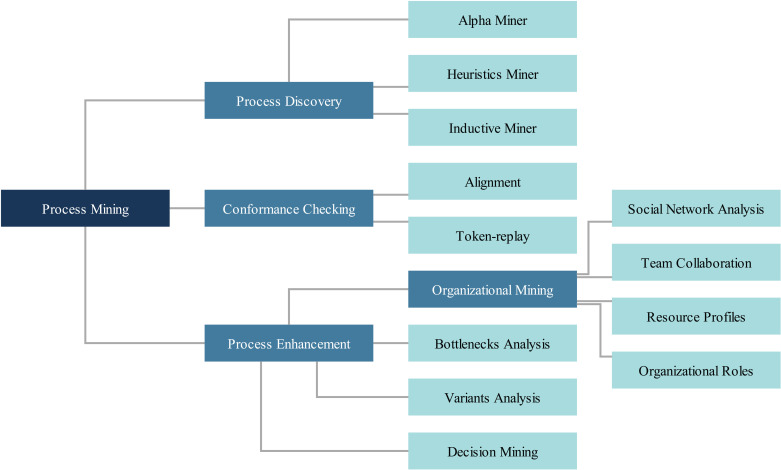
The process mining types and algorithms discussed in this paper.

In the following subsections, we will outline the fundamentals of these concepts, focusing on practical applications rather than mathematical intricacies. Readers are encouraged to consult the referenced literature to understand the underlying mathematics [10,12,24]. The subcategories of the four main process mining types are structured hierarchically in Fig 2.

Following the introduction of the algorithms in this section, we will detail the methods, including data preparation, algorithms, and the software package used for analysis, in section 2. In the results section (section 3), we will explore the three main types of process mining through complementary examples, elucidating the concepts shown in Figs 1 and 2. The discussion, section 4, will leverage organizational mining to bolster the argument for a change in leadership style as a prerequisite for efficient process mining implementation. Finally, we will present the conclusions in section 5.

### 1.1 Event data

Event data [12] is the foundational input for process mining, which is used to structure an event log. An event log (*L*) is a multiset of traces, where each trace *σ*_*i*_* *= ⟨*a*_1_*, a*_2_*,..., a*_*k*_⟩ is a sequence of activities (*a*_*i*_). Each activity *a*_*i*_ represents a discrete event in the process.

*σ*_*i*_: A sequence of activities representing one process instance. *L*(*σ*_*i*_): The number of occurrences of trace *σ*_*i*_ in *L*. An event log, for example, can be visualized as follows:


L = {⟨a1, a2, a3⟩, ⟨a1, a3⟩, ⟨a2, a3, a1⟩}

In this context, two fundamental relations are defined:

(1) **Directly-follows relation** (≻): *a*_*i*_* *≻ *a*_*j*_ if activity *a*_*i*_ is directly followed by *a*_*j*_ in at least one trace.
(2) **Parallel relation** (∥): *a*_*i*_ ∥ *a*_*j*_ if *a*_*i*_ and *a*_*j*_ can occur concurrently in at least one trace.

In practice, the starting point for process mining is an event log obtained from event data. Each event in the event log corresponds to an *activity* (i.e., a step or task in a process) with a *timestamp* (usually date and time) and is linked to a specific *case* (i.e., a patient number). The simplest form of an event log includes three essential columns: Case ID, Activity, and Timestamp [8]. These columns are the minimum requirements for any process mining task. These columns form the foundation of any process mining analysis:

- **Case ID**: This column identifies and differentiates individual cases or instances within a process. It serves as a unique identifier for each case and helps track and analyze a specific case’s life cycle.
- **Activity**: This column captures the specific actions or tasks performed within the process. It provides a detailed record of the activity sequence, allowing for analysis of the process flow, frequency of activities, and any potential bottlenecks or deviations.
- **Timestamp**: This column records each activity’s time and date. It enables the analysis of the chronological order of events and duration of activities and helps identify time-related patterns and trends within the process.

While the core columns explained above are essential, additional attributes can provide deeper insights into the process:

- **Resource**: It clarifies who performs each activity and what equipment or systems are being utilized, identifies the workload distribution, and analyzes performance based on roles, serving as the fundamental input for organizational mining.
- **Technique, modality, etc.**: These particular attributes in radiation oncology provide context for detailed analysis, including variations in decision-making and root cause analysis in process execution based on the technique used.

Further details on the parameters derivable from the event log are delineated in S1 Appendix. To demonstrate, we present several rows from an event log in Table 1, applying color-coding to the attribute previously discussed. A summary of various statistics derived from this event log is presented in Table 2. Regardless of the software packages utilized for data analysis, these packages typically offer a comprehensive statistical overview of the data upon import. Case duration analysis, summarized in Table 2, revealed insights into the overall process timeline. Across all 538 cases, which were all classified as’completed’ within this dataset, the mean case duration was 42.3 days, with a median of 15 days and a range spanning from 0 to 283 days (SD = 64.4 days). These statistics, presented in days, address the requested detail on case duration metrics, providing a comprehensive overview of the typical and variable timelines observed within the analyzed event log.

**Table 1:** The minimum requirements for an event log include *Case ID*, *Activity*, and *Timestamp*. Additional information, such as *resources*, helps understand workload distribution and role-based performance, and other attributes enrich the analysis.

Case ID	Activity	Timestamp	Resource	Technique	Modality
case 2	Treat	2023-01-10 03:07:00	Therapist	VMAT	EBRT
case 1	Plan	2023-01-03 19:02:00	Dosimetrist	3D	EBRT
case 3	QA	2023-01-07 03:51:00	Physicist	VMAT	EBRT
case 19	Treat	2023-01-06 11:51:00	Therapist	HDR	Brachy
case 2	Consult	2023-01-02 17:15:00	Physician	VMAT	EBRT
case 1	Record	2023-01-07 19:51:00	Nurse	3D	EBRT

**Table 2:** Summary of key event log statistics.

Statistic	Summary
Number of Cases	538 (All cases in the dataset are considered ’completed’ based on activity count criteria)
Frequency of Activities	Physics Chart Check (851), Contour OARs (669), Import CT (666), Create Plan (660), Contour Target (659), Plan Review (650), Complete Plan for Treatment (650), Therapist Chart Check (642)
Case Duration	Mean: 42.3 days, Median: 15 days, Range: 0–283 days (SD: 64.4 days)
Throughput Time	From 2023-06-05 13:18:11–2024-06-03 13:43:23
Activity Duration	Complete Plan for Treatment: 360.09 days, Contour OARs: 358.09 days, Contour Target: 358.34 days, Create Plan: 360.12 days, Import CT: 358.09 days, Physics Chart Check: 362.86 days, Plan Review: 360.09 days, Ther- apist Chart Check: 364.02 days
Start and End Activities	Start: Import CT (530), Contour Target (3), Contour OARs (3), Therapist Chart Check (2), End: Therapist Chart Check (465), Physics Chart Check (64), Contour OARs (5), Create Plan (4)
Resource Utilization	Dosimetrist (2645), Physician (1309), Physicist (851), Therapist (642)
Technique Distribution	IMRT (1958), 2D per plan (1765), 3D per plan (1452), electron (168), SBRT/VMAT (24), VMAT-Rapid Arc IMRT (24), en face (16), Field in field (16), 3D Tangents (8), VMAT (8), 3 D (8)
Resource Performance	Dosimetrist: 361.02 days, Physician: 360.14 days, Physicist: 362.86 days, Therapist: 364.02 days

### 1.2 Process modeling representations

Process modeling representations provide structured ways to represent discovered processes [10,24,25]. Common representations include Petri Nets, a mathematical modeling language for concurrent systems; Business Process Model and Notation (BPMN), a graphical standard for business process modeling; Directly-Follows Graphs (DFG), which visualize sequences of activities; Process Trees, hierarchical representations of process executions; and Heuristic Nets, which employ heuristics to depict complex systems. This manuscript emphasizes the practical application of process mining; hence, the detailed explanation of these notations has been relocated to the S2 Appendix.

**Fig 3:**
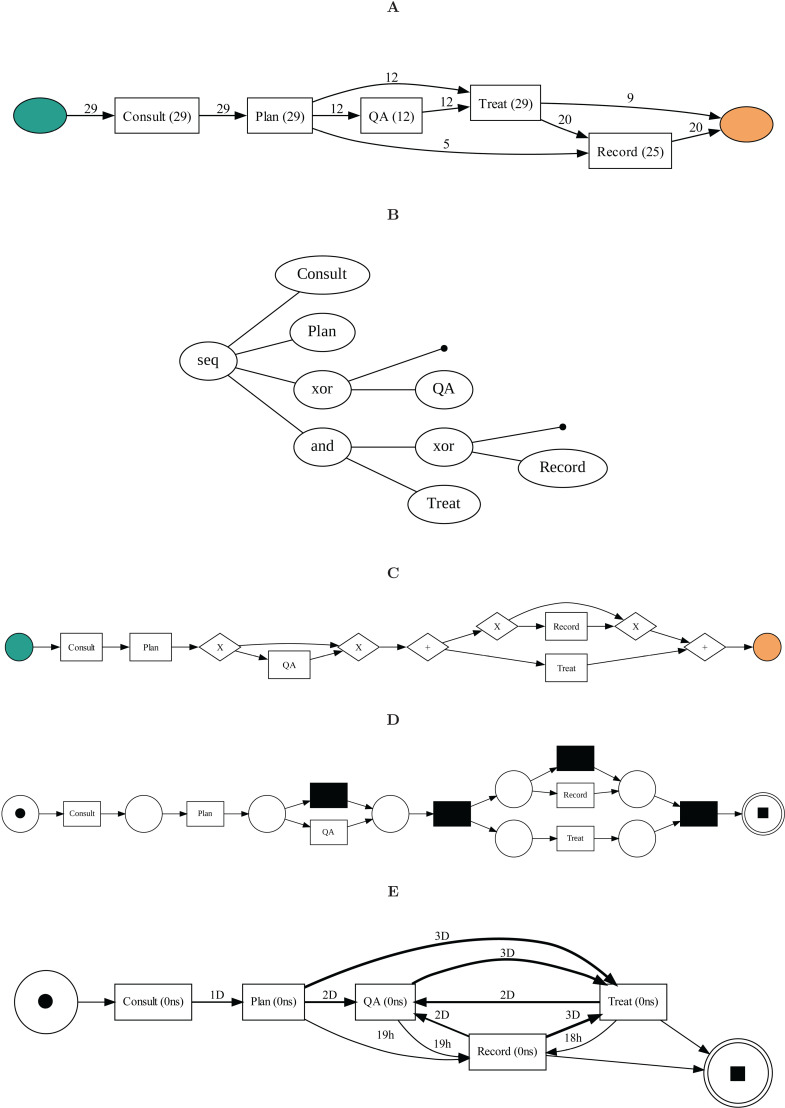
Visual representations of the event log using different notations and process discovery algorithms: A) Heuristics Net, B) Process Tree, C) BPMN, and D) Petri Net, E) DFG.

An example of a process visualized using the mentioned five methods is shown in Fig 3. For simplicity and readability, particularly for practitioners in the oncology domain, this manuscript primarily utilizes DFG and heuristic net visuals due to their intuitive nature. An example of a DFG visual is given in Fig 3E. This DFG model is drawn using a performance variant that indicates the duration of each activity. The ”0 ns” in the nodes indicates no measurable delay between completing one activity and the start of the next. This may be the case in the workflows of radiation oncology EMRs due to the triggering of tasks immediately upon completing the preceding tasks. While simpler representations like DFGs enhance readability, it’s important to note that more complex representations like Petri nets and process trees are invaluable for back-end computational purposes such as conformance checking and decision-making.

### 1.3 Process mining algorithms

In Radiation Oncology, clinical and administrative workflows are modeled as processes, with each patient case representing a sample. Process discovery creates a process model from these instances, balancing capturing real behaviors and not allowing unrelated ones. We focus on three key algorithms: Alpha Miner, Inductive Miner, and Heuristic Miner. A brief description is given here, and more details on the algorithms are provided in S3 Appendix. Although we explained DFG algorithm in S3 Appendix, DFG is usually considered as a representation rather than an algorithm for mining.

#### 1.3.1 Alpha miner.

The Alpha Miner generates a Petri net model from event logs by capturing directly-follows and parallel relations between activities. This foundational algorithm provides a basic representation of the process structure (more details in S3 Appendix).

#### 1.3.2 Inductive miner.

The Inductive Miner generates hierarchical process models by identifying repetitive patterns within traces. It handles noise and incomplete data effectively, making it a robust choice for process discovery (more details in S3 Appendix).

#### 1.3.3 Heuristic miner.

The Heuristic Miner balances simplicity and robustness by handling noise and infrequent behavior in event logs. It constructs a Heuristic Net that captures essential process behavior while being resilient to irregularities (more details in S3 Appendix).

### 1.4 Conformance checking

Conformance checking assesses how well an event log aligns with a process model, identifying discrepancies and measuring fitness, precision, and other metrics. We describe two key techniques below with simple explanations of algorithms given in S4 Appendix.

#### 1.4.1 Token-based replay.

Token-based replay replays the event log on the process model to identify deviations and measure fitness. It uses tokens to track the execution of traces within the Petri net, noting where tokens are missing or remain (details in S4 Appendix).

#### 1.4.2 Alignments.

Alignments compare traces with the process model by finding the optimal alignment balancing synchronous moves, log moves, and model moves. This technique calculates conformance metrics based on the alignment, providing precise insights into deviations (details in S4 Appendix).

**Conformance checking metrics:** In process mining, conformance checking involves evaluating the alignment between an observed process (captured in an event log) and a predefined process model. This evaluation uses several key metrics, each providing unique insights into the model’s quality. The primary metrics used in conformance checking are fitness, precision, generalization, and simplicity. Below is a summary of these metrics [26]:

**Fitness:** Fitness measures the proportion of observed behavior in the event log that the process model can accurately replay. It indicates how well the model represents the actual process. A high fitness score suggests that the model fits the observed process well, with minimal discrepancies. Conversely, a low fitness score indicates that the model fails to capture significant parts of the observed behavior.

**Precision:** Precision assesses the extent to which the process model does not allow for behaviors not observed in the event log. It helps prevent the model from being overly general and allowing too much variation. High precision indicates that the model matches observed behavior closely without accommodating extraneous paths or activities. Low precision suggests that the model might be too permissive, allowing for behaviors not supported by the event log.

**Generalization:** Generalization evaluates the model’s capacity to generalize beyond the observed behavior, capturing the underlying process in a way that can handle new and unseen instances. A well-generalized model should be robust enough to handle variations in the process that were not present in the event log. Poor generalization means the model is too tailored to the observed instances and might perform poorly with new data.

**Simplicity:** Simplicity measures the complexity of the process model. It reflects how straightforward and comprehensible the model is, balancing the need for accuracy with the desire for lucidity. Simple models are preferred for their ease of understanding and maintenance. A model that is too complex might capture the observed behavior accurately but at the cost of being difficult to interpret and use.

These metrics comprehensively evaluate how well the process model aligns with the event log, balancing the need for accuracy, simplicity, and robustness.

### 1.5 Process enhancement

Process enhancement in process mining aims to improve the efficiency and effectiveness of business processes by analyzing event logs and identifying opportunities for optimization. This section discusses various techniques and strategies used in process enhancement, focusing on uncovering bottlenecks, reducing cycle times, and improving overall process performance.

#### 1.5.1 Bottleneck analysis.

Bottleneck analysis identifies stages where delays occur, causing a slowdown in overall process performance. Organizations can implement targeted interventions to alleviate congestion and streamline process flow by pinpointing these bottlenecks. Here, analyzing case duration helps identify long-running cases and activities contributing to extended cycle times. A performance DFG or a heuristic net is applicable here as it highlights the average time between activities, helping to identify where delays occur.

#### 1.5.2 Variant analysis.

Variant analysis examines different paths (variants) that cases take. Understanding these variants helps identify common patterns and deviations, providing process standardization and improvement insights. Analyzing the top variants that cover a significant portion of the cases helps to focus on the most frequent process paths.

#### 1.5.3 Decision mining.

Decision mining is a powerful technique within process mining that leverages the rich event data captured during process execution to gain valuable insights and support decision-making. By applying machine learning algorithms to event logs, decision mining enables the extraction of knowledge about how choices made within a process influence its outcomes [27]. This knowledge can improve the process’s efficiency, effectiveness, and compliance. Two key applications of decision mining stand out:

**Operational Decision Support:** In this scenario, decision mining aims to assist decision-makers in real-time as they navigate a process. The technique can identify patterns and correlations that predict the impact of various choices or actions by analyzing historical event data. This predictive information can guide decision-makers toward optimal actions, leading to better outcomes and improved performance.

**Root Cause Analysis and Process Optimization:** This application of decision mining focuses on understanding the underlying factors contributing to specific process outcomes, whether desirable or undesirable. By examining how decisions and events influence outcomes such as cycle times, costs, or error rates, decision mining can reveal the root causes of inefficiencies or problems. This knowledge empowers organizations to make informed changes to the process, leading to optimizations and improvements in overall performance.

#### 1.5.4 Organizational mining.

Organizational mining focuses on understanding the interactions, roles, and performance of resources within an organization based on event log data. This section covers key techniques and metrics used to analyze organizational dynamics. Social Network Analysis (SNA) aims to understand resource interactions and communication patterns. By visualizing and analyzing social networks derived from event logs, SNA provides insights into the collaboration and communication structure within the organization. Using SNA, the following items can be classified:

**Organizational Roles:** Within a process, a role represents a set of activities consistently performed by a particular group of resources. It defines a specific function or responsibility within the organization. Grouping activities into roles provides valuable insights into task allocation and resource utilization. By understanding which activities are typically executed by which roles, we can better understand the division of labor within the organization. Analyzing roles helps identify potential bottlenecks, optimize resource allocation, and improve overall process efficiency.

**Resource Profiles:** Creating resource profiles involves aggregating performance data and analyzing resources’ efficiency and skill sets. This helps in understanding the strengths and areas of improvement for individual resources. Key metrics could include Workload, Performance efficiency, Task variety, and Resource utilization.

**Team Collaboration:** Team collaboration is a critical aspect of any organizational process [28]. It encompasses how teams interact, share information, and coordinate efforts to achieve common goals. Understanding these dynamics can highlight successful collaboration patterns, reveal potential communication barriers, and identify opportunities to streamline workflows and improve efficiency. Critical factors in team collaboration analysis include:

**Interaction Frequency:** High interaction frequency, such as working together on the same case, can indicate strong collaboration. However, assessing whether these interactions are productive and contribute to positive outcomes is essential.

**Team Efficiency:** Analyzing team efficiency by examining the frequency of handing over work between team members helps identify potential bottlenecks or areas where collaboration could be improved to expedite processes.

**Cross-Departmental and Cross-Functional Interactions:** Understanding how teams from different departments work together, including the frequency and success of delegating tasks to other teams (subcontracting), can unveil opportunities to break down silos and improve communication. Analyzing the effectiveness of cross-functional projects can shed light on factors that contribute to successful collaboration between teams with diverse skill sets, leading to more efficient processes and better outcomes.

## 2 Methods

### 2.1 Event log

**Fig 4:**
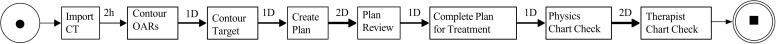
Process model planned (imagined) for the clinical case in the manuscript.

We utilized the Electronic Medical Record (EMR) system to generate an event log recording a year of treatment planning steps, from CT simulation to therapists’ checks before treatment. In this single-institution study, we leveraged data primarily from the ARIA Electronic Medical Record (Varian Medical Systems) and the Eclipse Treatment Planning System (Varian). While the methodology is broadly applicable, each site may need to adapt the data-extraction process depending on their EMR/TPS. In an ideal case, the department workflow is designed to follow eight activities for this process, summarized in Fig 4. Table 3 summarizes the event log attributes. As discussed in previous sections, three attributes are required to perform process mining: case ID (here, Patient ID), Activity, and Timestamp. The other attributes are optional but enrich the analysis by providing additional context and details.

**Table 3:** Data attributes overview.

Attribute Name	Type	Data Type	Level	Count
PatientSer	Case	String	Case	617
Technique	Other	String	Case	12
Activity	Activity	String	Event	8
End Time	End	Date	Event	5910
Site	Other	String	Case	115
Resource	Resource	String	Event	5
Start Time	Start	Date	Event	5910

### 2.2 Analytics

More than forty software packages and tools are available for process mining [12]. Among these tools, we employed an open-source Python software package called PM4Py [29]. Using the tools available, we cover most of the items presented in the introduction, such as statistical insights from the event log, process discovery, and conformance checking. We present a combination of process enhancements derived from process mining techniques and quality management methodologies, including brainstorming, the five whys, and a fishbone (Ishikawa) diagram for the process improvement segment. These quality management tools and methodologies are well-documented in existing literature, providing a robust framework for problem-solving in healthcare [4,30,31]. We aim to demonstrate how these tools seamlessly integrate with process mining, enhancing each other’s effectiveness within a well-designed quality management system.

### 2.3 Quality management

**Fig 5:**
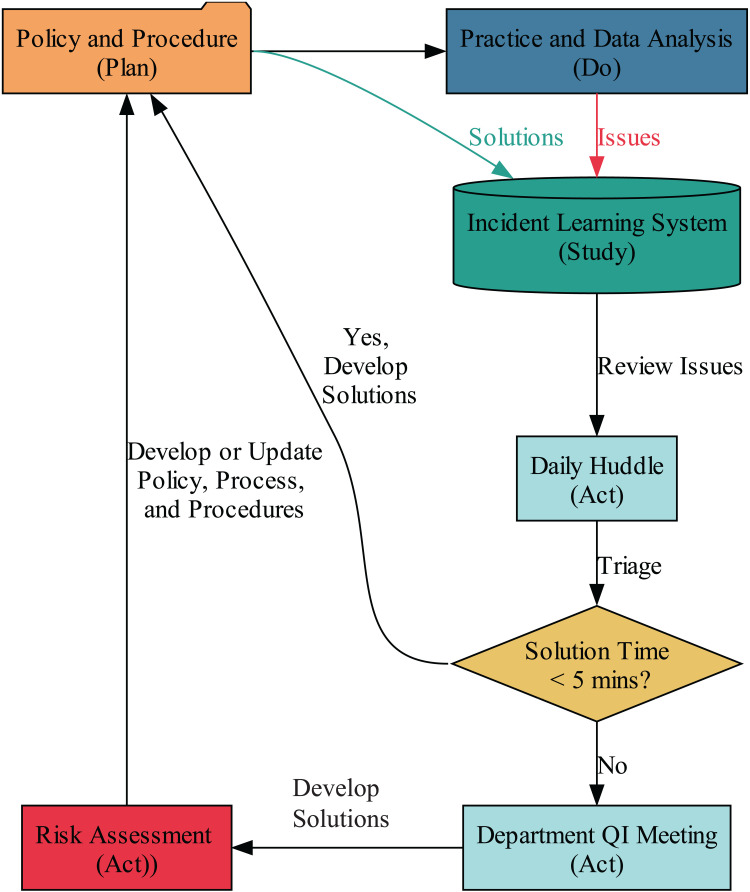
The department’s daily management system, a part of a broader quality management system, ensures a seamless flow of information between all components based on the guidelines [ 4,42].

Process mining proves beneficial if implemented into a broader system of quality management. A sub-process of the department’s quality management system relevant to this study is shown in Fig 5 [4]. The system is designed to facilitate a seamless flow of information. Policy and procedure (checklist) documents are readily available through the department’s incident learning system (ILS), which is network-accessible and mobile-friendly [2]. The ILS is a core element, heavily dependent on employee input [32]. Numerous studies and guidelines indicate that the more errors reported into the ILS, the less harm reaches patients [14,32–34]. Therefore, employees are often reminded that the objective is not merely to prevent error, but to prevent harm. The leadership’s role is to establish a culture of safety in which psychological safety for reporting errors is guaranteed [35,36].

This system allows employees to report problems quickly using the 5 Ws method [37]. The 5 Ws stand for When the issue was discovered, What the issue is, Where it occurred, Who detected it or Who affected by it (not who caused it), and Why it is considered an issue. The system dynamically evaluates whether a problem has been previously reported and addressed. If a problem occurs once, it is considered an isolated event. However, if it happens again, it is recognized as a pattern, flagging it for rigorous attention during QI meetings [38]. Recurrent issues, patterns, indicate an inefficient or premature solution that requires addressing its systemic root cause [39]. Reported problems are automatically displayed on the huddle’s electronic dashboard, integrated into the daily management system [40,41]. The ILS software lists the most relevant policies and procedures based on incident keywords. These policies must be reviewed and modified according to the root cause analysis results and developed interventions [4].

Issues reported during the preceding day are quickly triaged based on their difficulty level to solve, following Joint Commission recommendations [42]. The triage ends up with the problems being either addressed during the daily huddle or scheduled for review in the department’s periodic QI meetings. For example, a missing signature by a physician on a plan being treated today doesn’t require a change process and can be addressed immediately; however, if the ILS shows a pattern of missing signatures (recurrences), then that needs to be root caused in the QI meeting. Systems thinking suggests that if something occurs once, it’s an event; if it occurs repeatedly, it’s a pattern, and thus the system should be adjusted accordingly [38]. The decision-making chart of the department’s daily management system is illustrated in Fig 5. Solutions are logged back into the ILS to monitor efficiency and recurrence. By documenting solutions in the ILS, the team ensures daily awareness of the issues (situational assessment) [43]. The impact of these interventions will be assessed through subsequent continuous improvement efforts, as shown in Fig 5. Huddles and QI meetings fulfill the cognitive diversity criteria by ensuring at least one member from each discipline is present [44]. Cognitive diversity necessitates the fulfillment of psychological safety criteria.

Process mining is utilized not just for pinpointing problems but also for discovering best practices. Quality improvement systems are designed not only to address problems but also to analyze the processes that are performed correctly [45]. This process includes analyzing the event log for lean issues, conducting variant analysis with a Pareto diagram to identify the top variants that account for over 80% of cases as best practices [46], and performing conformance checking to uncover the least compliant cases concerning the planned process. A Pareto diagram is a bar chart representing the frequency or impact of problems or causes in descending order, helping identify the most significant factors in a dataset [37].

During the huddle or QI meetings, problems are categorized into three levels: simple, complicated, and complex. This classification is essential because it requires distinct approaches for each category. Each level requires a tailored approach for effective resolution [28,47]. Table 4 provides the decision criteria for identifying whether a situation is complicated or complex.

**Table 4:** Decision criteria for identifying complex or complicated situations [47].

Criteria	Yes (complicated)/ No (complex)
Has the problem been solved and implemented successfully in similar situations?	□ Yes (complicated) □ No (complex)
Does the problem have clear cause-and-effect relationships?	□ Yes (complicated) □ No (complex)
Can a checklist be used to solve the problem?	□ Yes (complicated) □ No (complex)
Is there an expert available who has previously solved the problem?	□ Yes (complicated) □ No (complex)
Is there an existing packaged solution available for the problem?	□ Yes (complicated) □ No (complex)
Are there only a few unknowns or uncertainties associated with the problem?	□ Yes (complicated) □ No (complex)

#### 2.3.1 Simple issues.

A *simple* brainstorming session often addresses problems following a direct cause-and-effect relationship. During this brainstorming, team members freely generate ideas without judgment, fostering creativity and innovation.

#### 2.3.2 Complicated issues.

For *complicated* problems that still follow a linear cause and effect but need to be investigated more deeply, we utilize the ”5 Whys” technique. This method asks ”why” to delve deeper into the underlying causes. A round of brainstorming follows each answer to a ”why” question to explore the reasons behind it. This iterative process continues until the fundamental issue within the system boundary is uncovered, ensuring a thorough examination of all potential causes. Although the method is called Five Why, the number of Whys asked depends on how fast the team converges to a solution or until the answers reach the system’s boundaries. For example, if continuing Whys ends up in the economic condition in the country, there is nothing the team can do about it, meaning that the system’s boundaries are crossed. In these cases, having the infrastructure for a complex adaptive system that responds to environmental change by adapting internally and maintaining stability is beneficial.

In addition to the ”5 Whys” technique, we also use the Ishikawa (fishbone) diagram for complicated issues. This method utilizes 5 whys in different categories visually represents the relationships between the causes and the problem, helping to identify and organize potential causes systematically.

#### 2.3.3 Complex issues.

*Complex* problems, those caused by different factors interacting with each other, demand a more comprehensive approach, combining brainstorming, the ”5 Whys” technique, and the Ishikawa diagram. These problems require multiple sessions of these methodologies to explore all potential causes thoroughly. The findings are then organized into an Ishikawa diagram, which visually represents the relationships between the causes and the problem. The 5 Whys and Ishikawa diagrams can help solve the majority of issues in a department. However, for a few percent of the complex issues, using complex problem-solving techniques such as causal loop diagrams (CLD) is necessary [48]. This diagram helps identify patterns and interconnections that are not apparent through linear analysis alone.

Whether simple or complicated, issues may be classified similarly due to the resemblance in their resolution approaches. Consequently, a matrix has been provided to swiftly determine these issues’ category, as shown in Table 4 [47].

Leaders intentionally speak last by accepting the team’s solution and promising to handle possible administration barriers, allowing team members to express their ideas and perspectives freely and ensuring that the brainstorming process is unbiased and inclusive [49].

### 2.4 Risk management

Given the inherent risks associated with radiation oncology, properly managing process changes is crucial [50]. Complex systems like radiation oncology are inherently sensitive to initial conditions [6,45,51]. Even minor changes can produce ripples throughout the system, potentially leading to significant, unforeseen consequences. This phenomenon underscores the importance of robust change management and risk management strategies. If not carefully managed, minor modifications can escalate and result in catastrophic failures. Following any process alteration or the development of a new procedure, such as the implementation of AI contouring, the QI team conducts a risk assessment before clinical deployment, in line with TG-100 guidelines [3,52,53]. This aligns the system with HRO principles: preoccupations with failure, reluctance to simplify interpretations, sensitivity to operations, and commitment to resilience [1].

## 3 Results

This section covers the main applications of process mining in a quality management system.

### 3.1 Event log

**Fig 6:**
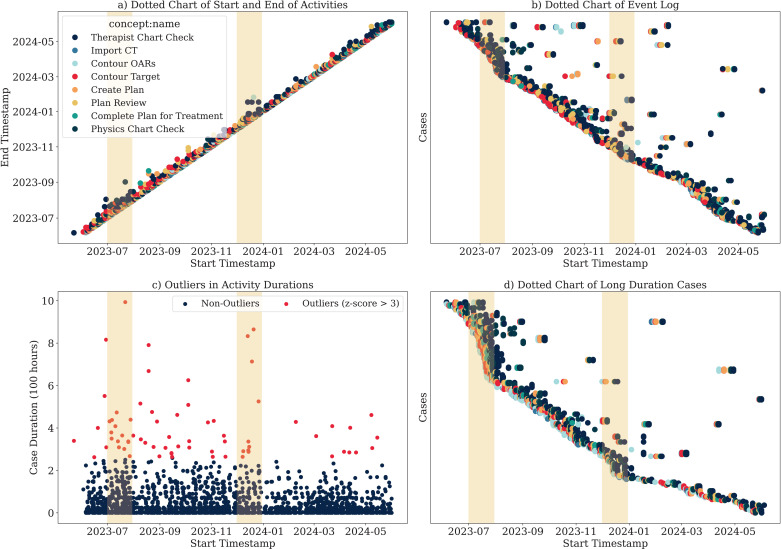
Even log summary: A) Start and end of each activity, B) Timeline of the activities, C) Identification of outliers using Z-score, e) Analysis of cases taking more than 14 days to complete.

Prior to delving into process discovery, this section demonstrates the value of a well-structured data log for gaining insights. Fig 6 presents the second part of the analysis of the event log data across four subplots, demonstrating the valuable insights that can be gleaned even before the process mining stage. Fig 6A, the dotted chart of the activities start and end visually represents the flow. These bursts of activities, shown by yellow bands, can be attributed to seasonal trends. Fig 6B provides a detailed dotted chart of the event log, mapping start times for case IDs. This visualization helps identify patterns such as the batching of cases or specific time frames where certain cases are initiated. The slopes in Fig 6B are regions of interest; steeper slopes indicate periods when multiple cases are triggered shortly, aligning with the high-activity periods seen in Fig 6A. In Fig 6C, we highlight outliers in activity duration. This plot is worth looking at as it identifies instances where activities took significantly longer than usual, which can point to potential bottlenecks or inefficiencies in the process that will be discussed shortly. Fig 6D displays a dotted chart of long-duration cases, highlighting those that fall into the top 5% of the duration. The correlation between these plots is obvious, with the timing of outliers identified in Fig 6D coinciding with the long-duration cases in Fig 6C.

### 3.2 Process discovery

The Heuristics Miner algorithm was applied to the event log data, generating a Heuristics Net to model the underlying process. Considering all observed process instances, the Heuristics Net is constructed from the complete event log. A Heuristics Net is a type of Petri net that incorporates heuristics to simplify and enhance the readability of the model B. These heuristics are rules or guidelines used in problem-solving or decision-making to achieve solutions more efficiently. These rules help reduce the complexity of the Petri net model, making it easier to analyze and comprehend the underlying process. The model can better represent the system’s essential characteristics by applying heuristics.

**Fig 7:**
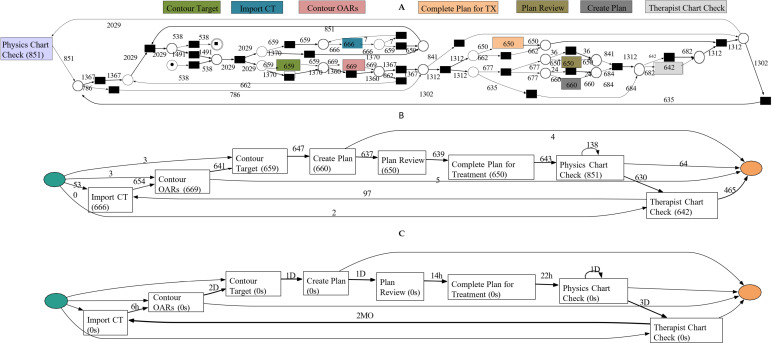
Process model (discovered) from the event log, A) Petri net of the discovered process, B) Frequency variant of Heuristic net of discovered process, C) Performance (duration) variant of Heuristic net of discovered process.

To demonstrate the advantages of heuristic net visualizations compared to Petri nets, Fig 7 presents the discovered model in both Petri net form and heuristic frequency and performance (duration) variants. In the duration-based model (Fig 7C), the tasks ”Contour OAR” and ”Therapist Chart Check” stand out with a duration of 2–3 days, respectively.

These longer durations suggest potential bottlenecks, as they appear significantly longer than other activities. This approach to identifying potential bottlenecks will be addressed further in the process enhancement phase, the subsequent step in the process mining methodology.

### 3.3 Conformance checking

In this study, we performed conformance checking D.1 of the data log using a token replay method to evaluate the fitness of each trace within our process model. The fitness metric provides insight into how well the observed behavior matches the modeled behavior, with a higher fitness indicating a closer alignment.

**Fig 8:**
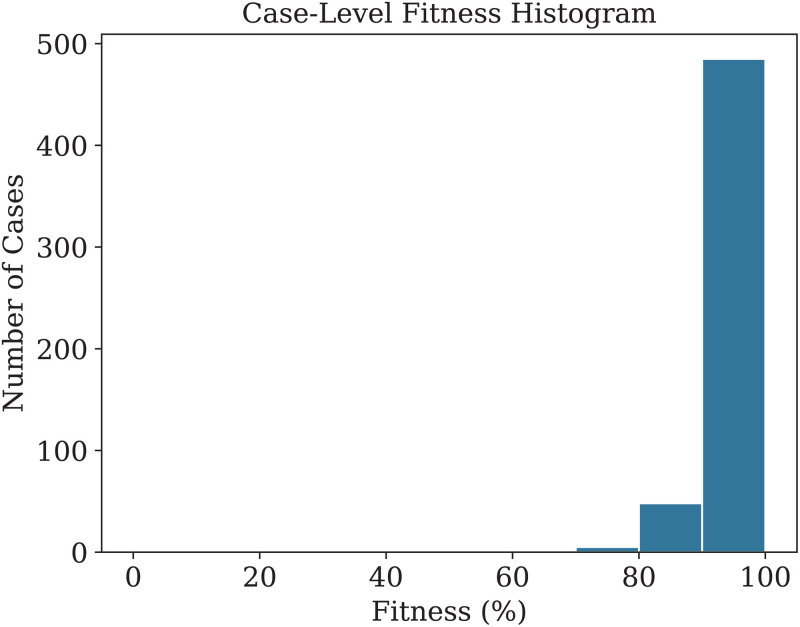
The histogram represents the fitness levels across all cases in the practical scenario, with most cases achieving full compliance (100% fitness).

The fitness scores were organized into a histogram comprising ten bins, as depicted in Fig 8. The study could progress by examining both extremes of the histogram: high fitness cases as exemplars of best practices, and low fitness cases as indicators of non-compliance. As high compliance cases were previously analyzed using a Pareto diagram, this section concentrates on the opposite spectrum, the low fitness cases, to provide a comprehensive view of the circumstances. The histogram indicates that the lowest bin holds a small number of cases, corresponding to traces with 60–70% fitness. This observation led to a more thorough examination of the processes linked to these low fitness scores.

Table 5 presents a comparison of conformance checking metrics using token-based replay and alignment algorithms, highlighting their respective strengths in terms of fitness, precision, generalization, and simplicity.

**Table 5:** Conformance checking metrics comparisons using two algorithms.

Metric	Token-based Replay	Alignments
Fitness	0.95	0.91
Precision	1.00	1.00
Generalization	0.96	0.96
Simplicity	1.00	1.00

### 3.4 Process enhancement

#### 3.4.1 Utilizing huddle for simple issues.

**Fig 9:**
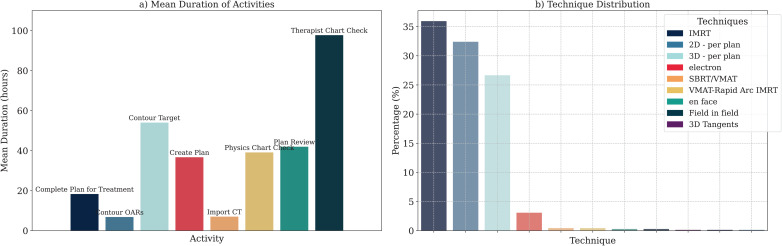
Analyzing the event log reveals that A) Therapist chart checks take a long time to complete, B) specific notations for treatment techniques are infrequently used and may be redundant.

Upon initial examination of the event log’s attributes and throughput, as shown in Fig 9, two critical issues were identified with apparent impacts on the process efficiency. The first issue was the extended time therapists spent checking the charts. Following the daily management system shown in Fig 5, the team quickly converged to a solution for this *simple* (section 2.3.1) issue during the daily morning huddle. Therapists revealed that they had intentionally delayed checking the plan until the day before the patient’s scheduled start of treatment. This prompt discussion led to the developing of a new project to optimize patient scheduling and therapists’ chart checks. The project aims to reduce the interval between CT simulation and treatment, preventing plans from remaining idle for extended periods after completion. Furthermore, as an immediate outcome of the discussion, therapists were advised and have agreed to prioritize reviewing plans as soon as they are available and when their workload permits. This proactive approach ensures timely feedback and allows sufficient time for the team to address any potential issues identified during the check, enhancing patient safety. The scheduling optimization project itself is still in progress.

#### 3.4.2 Brainstorming technique for simple-complicated issues.

**Fig 10:**
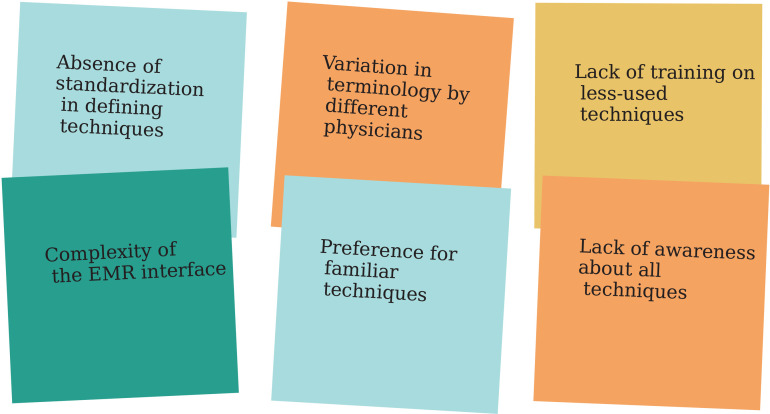
Brainstorming with sticky notes, whether paper or digital, helps prevent cognitive biases. This method is simple and easy, brainstorming; more advanced methods often build upon it.

The second issue was categorized as *complicated* (2.3.2). Analysis revealed twelve different terms for techniques used within the EMR. Our examination of the technique usage frequency, shown in Fig 9, indicated that eight techniques were rarely used and often unnecessary, causing inconsistency and inefficiency. Adopting a lean approach [54,55] to reduce the number of techniques was identified to improve efficiency and standardization. A quick brainstorming session using sticky notes addressed this issue without falling prey to cognitive biases. This method helped prevent biases by allowing all team members to contribute ideas on sticky notes, as shown in Fig 10. Accordingly, the multidisciplinary daily huddle participants identified the core problems as the absence of standardization in defining techniques and the different physicians’ terminology variations over time. These issues were exacerbated when physicians left the organization, resulting in fragmented data. The relevant policy document was examined and revised to ensure uniformity of terminology and technique application, especially during turnovers. We eliminated outdated technique labels and created a standardized list. The most significant outcome was reducing the options available to physicians, resulting in a more concise list to minimize possible human errors.

Following the implementation of an AI-powered contouring package, initial analyses indicated no significant reduction in task completion time. Despite this, dosimetrists reported a substantial improvement in their workflow and comfort levels due to the reduced need for manual contouring. Critically, our initial experience also highlighted the necessity of robust supervision and quality assurance protocols for AI-generated contours, a vital consideration reinforced by our TG-100 style risk assessment as detailed in Table 5.

#### 3.4.3 Bottleneck analysis.

**Fig 11:**
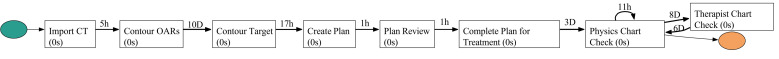
The process discovered for the case with the lowest fitness.

Upon examining the processes in this lowest fitness category, illustrated in Fig 11, we identified two activities that exhibited prolonged execution times: OARs contouring and the physics chart check. These delays were critical points of inefficiency within the workflow, warranting further analysis and targeted interventions, as will be discussed in a later section.

#### 3.4.4 Five whys methodology for simple and complicated issues.

**Fig 12:**
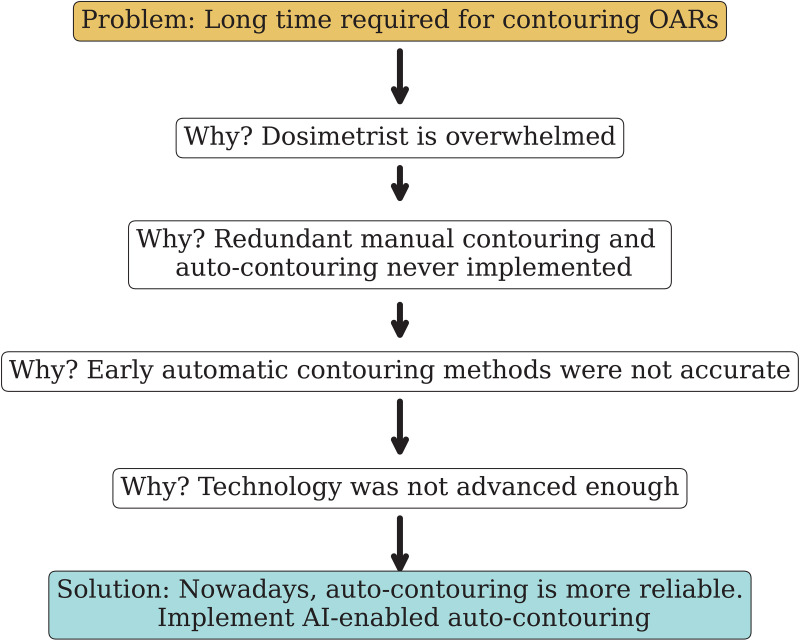
Root Cause Analysis using 5 Whys: first, clearly define the problem that needs resolution. Ask ”Why” several times: Question ”Why” the problem exists. For each response, continue asking ”Why” to delve further into the underlying cause. To guarantee thorough and impartial responses, conducting this exercise via brainstorming is advantageous (see Fig 10). While the technique is known as the five whys, the actual number of whys may vary depending on the speed of solution convergence or reaching the system’s limits.

The results of Fig 11 entered into the ILS as *complicated* issues. During the QI meeting, root cause analysis was conducted for the OARs contouring delays using the 5 Whys methodology. The root cause analysis results are presented in Fig 12. The root cause analysis revealed that the issue was not due to the performance of the dosimetrist but rather a systemic problem, which is mainly workload and lack of automation in the redundant portion of the contouring process. Automation has proven efficient in radiation oncology [56]. The delay in implementation was due to the inadequate accuracy of auto contouring in the past [57], and it was found that recent auto contouring products are within acceptance if supervised. Therefore, it was decided to implement an AI-based contouring package to optimize and expedite this process [58].

#### 3.4.5 Risk assessments for new processes.

**Fig 13:**
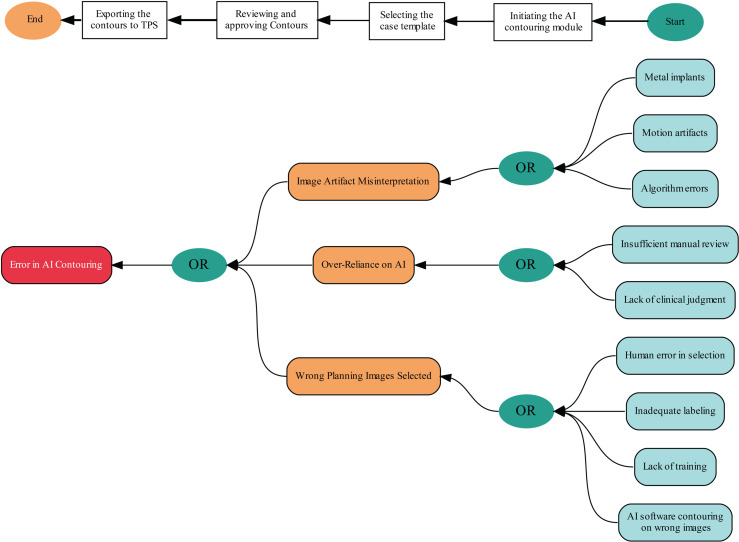
Process map (top) and Fault tree (bottom) produced from three failure modes in Table 6.

**Fig 14:**
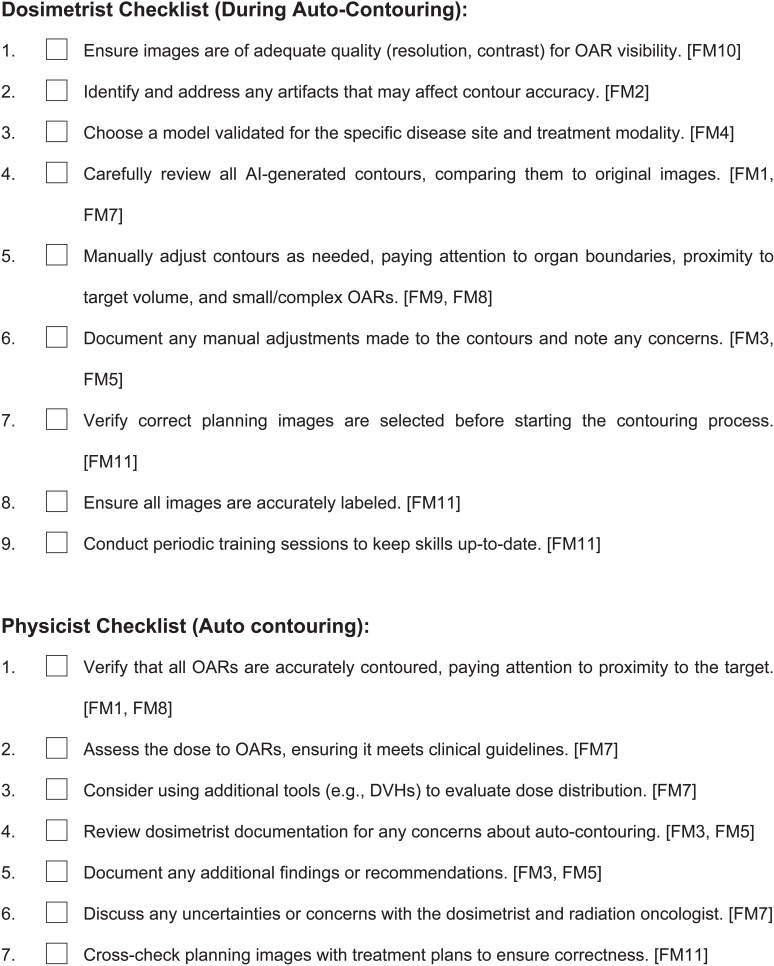
The checklist was developed to address top failure modes in Table 6. A practical checklist should follow certain rules such as certain fonts (San Serif), font size, number of items, etc [60].

The introduction of AI contouring necessitates a substantial modification and a thorough risk assessment by TG-100 [3]. The QI team evaluated the AI contouring software, where multidisciplinary team members pinpointed potential failure modes. Process mapping is the initial risk assessment phase, which provides an overview of the steps. The steps in the process map include: 1. Initiating the AI contouring module, 2. Selecting the appropriate case template, 3. Reviewing and approving the relevant contours, and 4. exporting the contours to TPS. Risk assessment is a proactive action to prevent potential issues and reduce risks. Table 6 lists not all but several failure modes for this process. The fault tree analysis for three failure modes is illustrated in Fig 13 along with the process map (top). In response to these failure modes, the team formulated two checklists following established standards [59,60], depicted in Fig 14.

**Table 6:** Failure mode and effects analysis (FMEA).

Failure Mode	S	O	D	RPN	Cause
Incorrect Organ Segmentation	4	3	2	24	Algorithm limitations, poor image quality
Image Artifact Misinterpretation	4	2	3	24	Metal implants, motion artifacts, algorithm errors
Software Integration Errors	3	2	4	24	Data transfer issues, compatibility problems
Lack of Validation in Specific Disease Sites	3	1	3	9	Insufficient testing on diverse patient data
Insufficient User Interface/Feedback	2	2	4	16	Poor design, lack of clear error messages
System Downtime or Malfunction	5	1	2	10	Hardware/software failure, network issues
Over-Reliance on AI	4	3	2	24	Insufficient manual review, lack of clinical judgment
Underestimation of OAR Proximity	4	2	3	24	Algorithm limitations, complex anatomy
Misidentification of Small OARs	3	2	3	18	Limited training data for small structures, low image contrast
Sensitivity to Image Quality	3	2	4	24	Poor resolution, noise, artifacts
Wrong Planning Images Selected	4	2	3	24	Human error in selection, inadequate labeling, lack of training, AI software contouring on wrong images

#### 3.4.6 Variant analysis.

**Fig 15:**
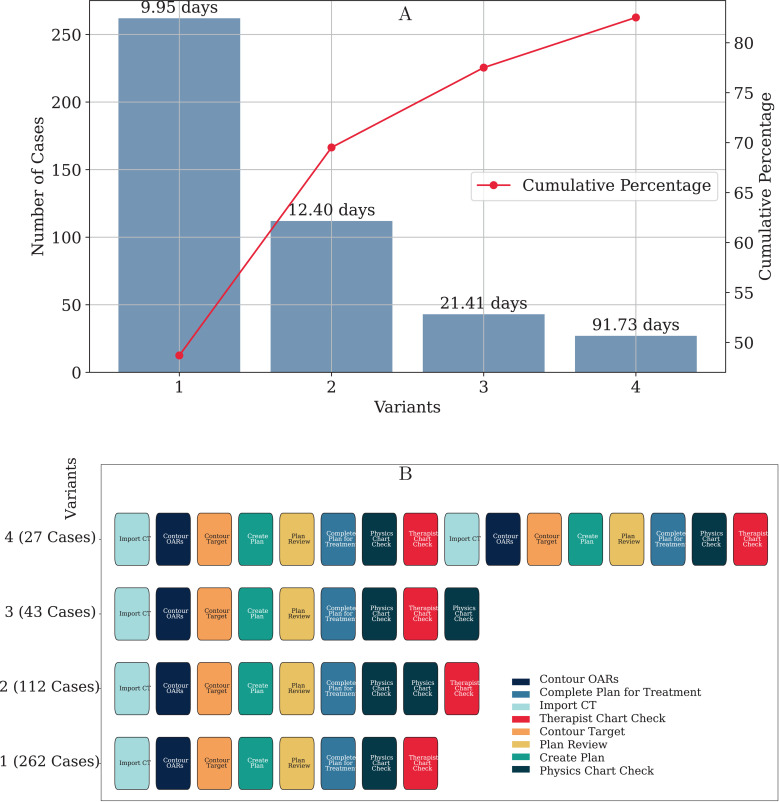
In this Pareto Diagram, out of many variants, four variants account for 80% of the cases. A) The numbers on the bars indicate the average duration of these variants, B) Top Variants.

Using process mining, further insights can be obtained by analyzing variants and creating a Pareto diagram. These are shown in Fig 15A and 15B. Although more than 44 variants were discovered from the log, the top 4 variants cover approximately 80% of the cases. This indicates that while a significant number of unique process paths, a few common paths dominate the workflow. Analyzing these predominant variants can help identify best practices and areas for standardization, leading to improved process efficiency.

**Fig 16:**
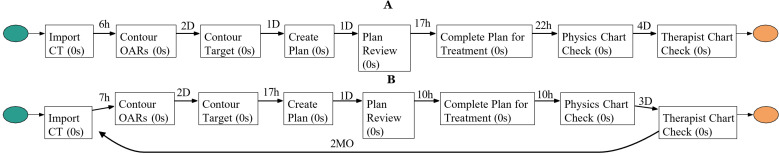
Discovered processes for variants 1 and 2: A) The process model was discovered using the heuristic miner algorithm for variant 1 in Fig 15, B) The process model discovered using the heuristic miner algorithm for variant 2 in Fig 15.

Fig 16A and 16B present the discovered processes for variants 1 and 2, respectively, using the heuristic miner algorithm. These detailed process models provide insights into the most frequent paths and their durations, enabling targeted interventions to streamline these high-frequency variants further.

#### 3.4.7 Ishikawa diagram for complicated and complex issues.

**Fig 17:**
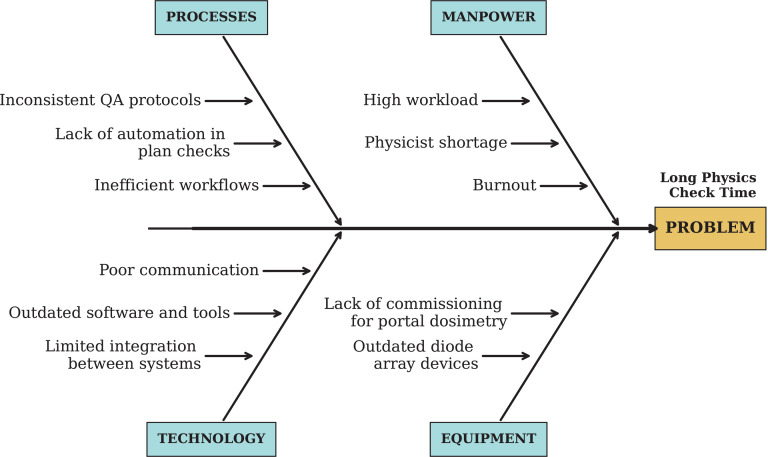
Utilizing an Ishikawa diagram for comprehensive root cause analysis encourages a broader exploration of potential causes beyond linear thinking by visually organizing and categorizing them into different groups.

For the delays in the physics check, the problem was categorized as *complex* (section 2.3), necessitating a more comprehensive approach. We employed an Ishikawa (fishbone) diagram to analyze the root cause, as shown in Fig 17. This method allowed us to systematically explore the various contributing factors, including manpower, equipment, processes, and technology. The analysis highlighted several areas for improvement, prompting targeted interventions to address these issues. The main interventions implemented are hiring a new physicist and implementing automation in chart check [61,62]. To assess the impact of the new physicist hire on the efficiency of physics chart checks, we analyzed the time taken to complete each check before and after the hire, while accounting for potential confounding factors such as the introduction of automation. Our analysis revealed a significant decrease in the mean time per check from 52.83 hours to 30.27 hours (p = 0.005) following the new hire. This improvement in efficiency, evidenced by a 43% reduction in time per check, underscores the positive effect of the new physicist on streamlining the chart check process.

#### 3.4.8 Interventions effectiveness.

Statistical techniques assist quality improvement teams in assessing the effectiveness of interventions. It’s important to demonstrate that the change in the system is not due to common causes but rather is the result of a specific intervention. Therefore, following the implementation of these interventions, we divided the data into two periods: before January 2024 and after. We observed a significant reduction from 15 to 10 days by comparing the average process throughput times. To validate that this improvement resulted from the interventions and not due to random variation, we conducted a t-test of the means. The statistical analysis confirmed that the reduction in throughput time was statistically significant, reinforcing the effectiveness of the implemented changes as seen in Table 7.

**Table 7:** Mean case duration before and after intervention (t-statistic = -3.35, p *< *0.001), indicating a statistically significant difference in means.

Time Period	Mean Case Duration (Business Days)
Before Jan 1, 2024	15.48
After Jan 1, 2024	10.29

**Fig 18:**
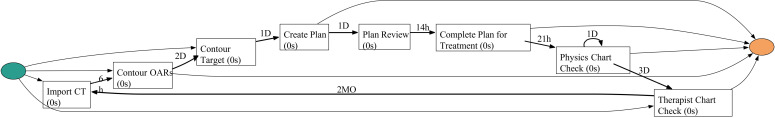
Process model discovered using the cases in top four variants (80%) of the cases according to Pareto Diagram 15A.

The process discovery of the event log after January 2024 produced the process model in Fig 18. This process model is mainly a product of the observed process behavior in the department. The department has several options: it can continue, make adjustments based on observed patterns, schedule activities and patient appointments accordingly, or work on improving and shortening the throughput time.

#### 3.4.9 Filtering low-frequency paths.

**Fig 19:**
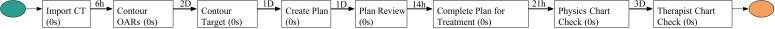
Process model discovered in 18 with low frequency paths filtered.

After implementing the interventions, the process model was further refined by filtering out low-frequency paths to focus on the most significant aspects of the workflow. This filtering helps in simplifying the model and highlighting the core processes. Fig 19 presents the process model discovered in Fig 18 with these low-frequency paths removed. This streamlined model allows for a clearer analysis of the main process flows, ensuring that attention is directed towards the most impactful areas for continuous improvement.

Regardless of the chosen path, continuous monitoring and recording of issues through the department’s incident learning system, huddles, and problem-solving via a robust continuous quality improvement program are essential [44].

#### 3.4.10 Decision mining.

**Fig 20:**
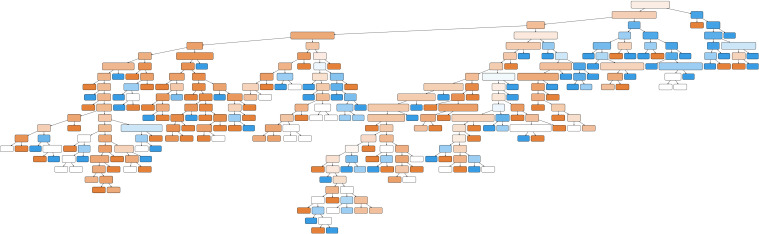
Decision tree for the entire event log. The text has been removed from the nodes to improve readability and allow the viewer to focus on the decision tree’s overall complexity.

**Fig 21:**
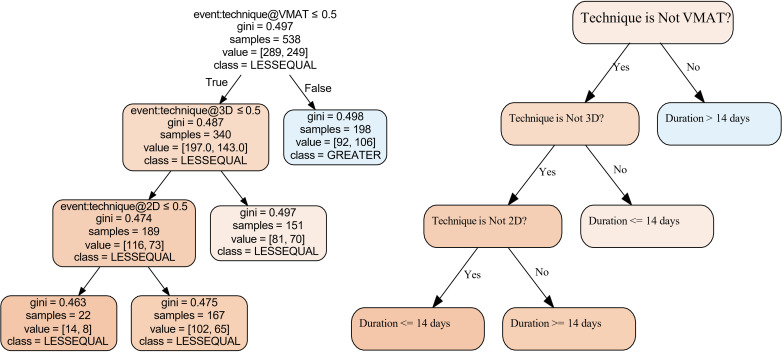
(Decision tree classifying cases based on whether their duration is 14 days or less, with the technique used (VMAT or other) as a splitting criterion. The Gini impurity measures how ”mixed” the classes are at each node. A lower value indicates a purer node (mostly one class). Samples indicate the number of cases reaching that specific node in the decision tree. Value shows the distribution of classes within a node. In this context, it represents the number of cases with duration <=14 days vs. > 14 days. The plot on the right represents our simplified interpretation of the decision tree.

**Fig 22:**
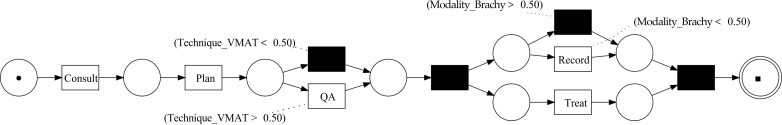
This decision-making and root cause analysis demonstration employs decision mining with machine learning and Petri Net, for example, in the manuscript, featuring annotated decision points. The net indicates that if the case is not VMAT (Technique VMAT *< *0.50), then QA was not conducted. Conversely, if the case is Brachy (Modality Brachy *> *0.50), the recording was not carried out, which presents an issue.

**Fig 23:**
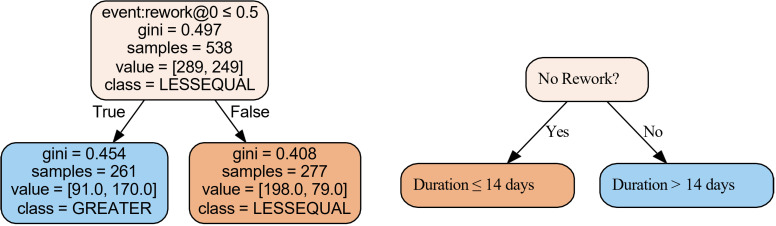
Process mining, combined with decision tree analysis using machine learning, can reveal insights into various process inefficiencies, such as rework. This dataset demonstrates that approximately 50% of cases involved some form of rework.

**Fig 24:**
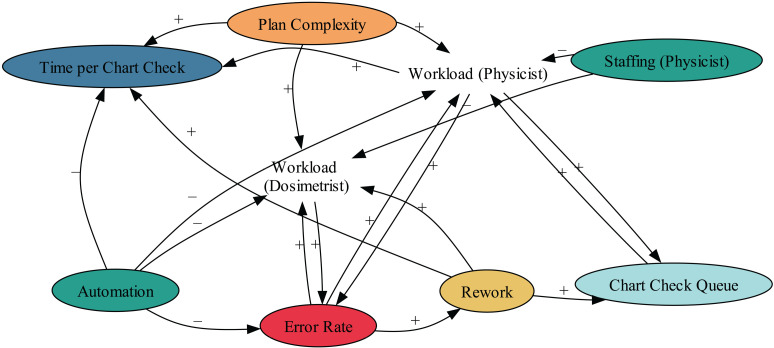
This causal loop diagram illustrates the interrelated relationships and feedback loops within a complex system, showing how alterations in one aspect can affect others, sometimes in unforeseen ways. A plus sign denotes a positive alteration, while a minus sign indicates a negative one. For instance, an increase in staffing leads to a decrease in the physics load.

A thorough analysis of decision mining from the log file, as depicted in Fig 20, uncovers significant insights that appear overwhelming. These decision trees are obtained using machine learning classification algorithms [29]. It is more effective to narrow the decision mining to specific states or cases for more manageable insights. For example, the analysis becomes more apparent by focusing on whether techniques take longer or shorter than 14 days to complete, as shown in Fig 21 accompanied by our simple interpretation on the right for readability. This figure indicates that the method used significantly predicts trace duration. Specifically, the VMAT (Volumetric Modulated Arc Therapy) technique has a trace duration exceeding 14 days. If VMAT is not used, the decision tree further splits based on the 3D and 2D techniques. For cases where neither 3D nor 2D are used (electron cases), the trace duration tends to be 14 days or less (Fig 22). These findings highlight the impact of specific techniques on process duration, suggesting that VMAT is linked to longer trace duration, while other techniques are associated with shorter duration. In this application of decision mining, the team can either continue with the existing process map or split it into two separate maps: one for VMAT cases and another for non-VMAT cases. This approach allows for more tailored process management and optimization based on each type of case’s specific requirements and characteristics. Linear thinking may lead one to believe that VMAT cases take longer due to their complexity, which is only partially correct. However, this is not the complete picture. For example, analyzing cases that exceed 14 days and constructing a decision tree based on reworks (where tasks are repeated at least once) unveils the results shown in Fig 23, adding another dimension to the analysis. When considered in its entirety, the complexity of the plan and the reworks might not be the only reasons for extended durations in complex systems. The interconnectedness, sensitivity to initial conditions, and the nonlinearity of these systems indicate that a mix of various elements and their interplay through feedback loops can influence the outcome. In this case, both the complexity and the reworks may interact with each other and with other system components. While linear approaches like brainstorming, the 5 whys, and Ishikawa diagrams work for most problems, truly complex issues may require a nonlinear system methodology, such as a causal loop diagram [48], that accounts for interconnectedness and feedback loops. An illustrative causal diagram of our discussion is presented in Fig 24, showing how different factors can influence each other via positive or negative feedback loops.

#### 3.4.11 Organizational mining.

**Fig 25:**
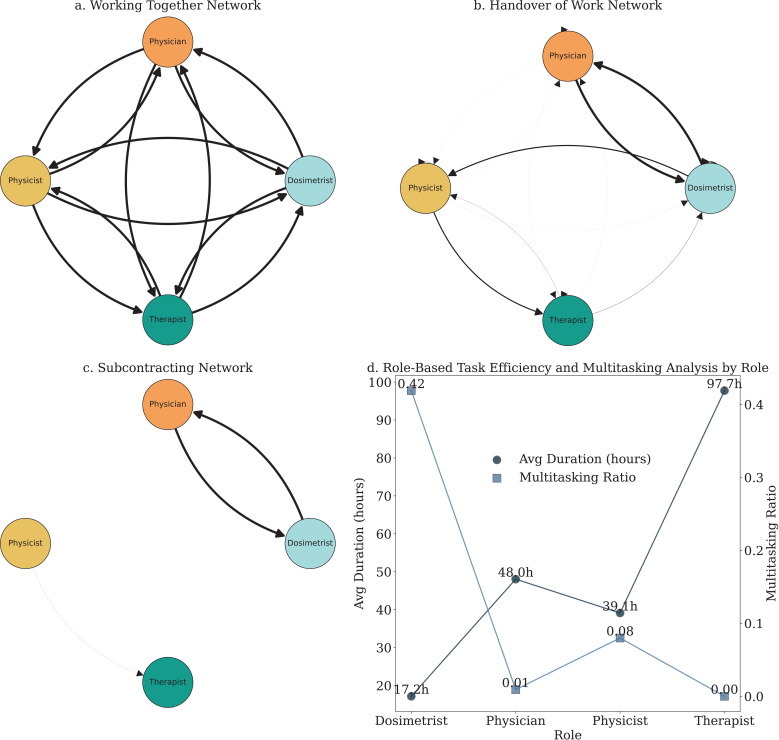
Network graphs depict the Handover of Work, Subcontracting, and Collaboration Networks. These visualizations underscore the interconnectedness and importance of information exchange and communication between resources. The thickness of the lines quantifies the intensity or frequency of communication.

Beyond the immediate process improvements discussed earlier, process mining also offers significant insights into organizational dynamics through organizational mining techniques. Fig 25 presents detailed analyses on various organizational mining aspects, further illustrating the transformation in leadership perspectives.

**Team Collaboration Networks:** The analysis of different collaboration networks reveals how teams interact within the organization. Fig 25a, 25b and 25c showcase the ‘Working Together Network‘, ‘Handover of Work Network‘, and ‘Subcontracting Network‘, respectively. These networks highlight the frequency and nature of interactions among team members, providing insights into collaboration patterns and identifying potential communication barriers, as discussed in the introduction.

**Role-Based Task Efficiency and Multitasking:** Table 8 presents detailed results of organizational mining algorithms for dosimetrists, including metrics such as distinct activities, activity frequency, completions, multitasking, and average duration.

**Table 8:** Results of organizational mining algorithms for dosimetrist.

Attribute	Value
Distinct Activities	4.0
Activity Frequency (Contour OARs)	0.2
Activity Completions	947.0
Case Completions	216.0
Fraction Case Completions	1.0
Average Workload	0.0
Multitasking	1.0
Average Duration Activity (Contour OARs)	40880.1 s
Average Case Duration	3043495.4 s
Interaction Two Resources (Dosimetrist and Physician)	216.0
Social Position	1.0

Fig 25d combines scatter plots of role-based task efficiency and multitasking ratios. By analyzing the average task duration and multitasking ratios for each role, we gain insights into resource utilization and identify potential bottlenecks. This detailed profiling helps in optimizing task allocation and improving overall process efficiency, aligning with the organizational roles and resource profiles described earlier.

These results highlight the importance of teamwork and leadership in optimizing organizational performance. The insights gained from organizational mining reveal how different roles interact and provide a comprehensive picture of workflow dynamics within the team. Such detailed analyses enhance collaboration, improve communication, and ensure efficient process management, reinforcing the points made in the introduction about the value of understanding organizational roles, resource profiles, and team collaboration.

## 4 Discussion

The field of radiation oncology is rapidly advancing, necessitating the development of efficient and adaptable processes. Integrating process mining into radiation oncology represents a significant advancement, offering detailed insights into clinical workflows and operational efficiencies. This discussion explores the broader implications of process mining, particularly its role in transforming leadership perspectives within the field.

Process mining leverages data from existing information systems to provide a granular view of clinical workflows. By analyzing event logs, process mining uncovers deviations from standard procedures, identifies bottlenecks, and highlights opportunities for improvement. This data-driven approach aligns with the increasing emphasis on precision medicine and personalized patient care in radiation oncology. However, to fully realize these advantages, a shift from traditional management to adaptive leadership is essential.

**Fig 26:**
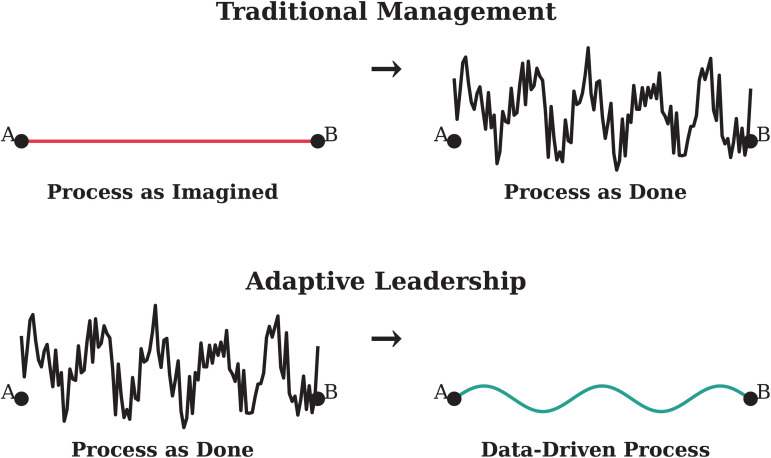
Contemporary leadership approaches are essential for navigating complex systems. Traditionally, policies and procedures are imposed top-down [4]. However, in the age of data, it’s imperative for processes to develop organically within the heart of the work environment, exemplified by process mining.

Fig 26 illustrates the difference between traditional management and modern leadership (specifically adaptive leadership among many modern leadership styles [63]), contextualized within the framework of process mining:

1 Process as Imagined or as Planned (Traditional Management)
In traditional management, clinical workflows are often predefined based on established protocols and best practices and are usually dictated top-down [4]. This approach can be rigid and may not fully capture the complexities of today’s processes. A rigid system is prone to safety issues as it usually fails to manage the unexpected [51]. This concept is fundamental to high-reliability organizations [64]. The static nature of predefined workflows can lead to inefficiencies and a lack of adaptability in responding to unique clinical scenarios.
2 **Process as Done** (Traditional Management)
Actual clinical practice often deviates from planned workflows due to patient variability, unexpected challenges, and emergent clinical decisions. Traditional management may struggle to reconcile these deviations, leading to gaps between planned and actual care delivery. This usually leads to violating the safety culture, blaming, and fear in the organization [5,35]. The inability to dynamically adjust to real-world conditions can compromise operational efficiency and patient outcomes [6].
3 **Process as Done** (Modern Leadership)
Modern leadership recognizes these deviations and uses data-driven insights to monitor behaviors and patterns in actual clinical practices. By analyzing real-time data, leaders can identify patterns and underlying causes of inefficiencies. This approach fosters a more adaptive and responsive leadership style, enabling continuous process improvements and better alignment with clinical realities. Studying the process as done has another important benefit that leaders can learn from the cases that went right rather than solely focusing on what went wrong [45].
4 **Data-Driven Process** (Modern Leadership)
The culmination of modern leadership is the adoption of data-driven processes. Leaders leverage process mining to gain insights into workflow performance, making decisions based on empirical evidence and real-time data. This method empowers leaders to make informed decisions, adapt swiftly to changes, and continuously refine workflows for optimal patient care.

The application of process mining in radiation oncology signifies a shift towards a data-driven leadership model. Process mining requires leaders to create an infrastructure (Fig 5) that enables teams to identify inefficiencies and improve autonomously. This infrastructure should promote four key principles: Transparency and Accountability, Adaptability and Resilience, Empowerment and Engagement, and Innovation and Improvement. A well-designed infrastructure facilitates smooth information exchange across various quality management system elements.

Although it may sound redundant, we emphasize the importance of risk assessment before making any changes in a process, regardless of the methods used to discover the required change [3].

### 4.1 Limitations

This study has several limitations:

- **Single Institution:** The data and outcomes reflect a single center’s workflows.
- **Workflow-Specific Results:** Results may not fully generalize to centers that have different methods or dedicated workflows to mitigate delays.
- **Software-Specific Processes:** We extracted event logs from ARIA/Eclipse, therefore, other TPS/EMRs may require different data mining pipelines.
- **Scope Restriction:** We focused on the planning workflow (CT to treatment start); future analyses could integrate more steps (machine QA, on-treatment verification, etc.).

## 5 Conclusions

Process mining can be a valuable tool for improving efficiency, quality, and decision-making in radiation oncology. By systematically collecting and analyzing event data, departments may uncover hidden workflow patterns, expedite bottleneck resolution, and adapt to evolving clinical needs. These insights enabled us to implement targeted interventions, improving throughput time and overall process efficiency.

Integrating process mining into radiation oncology quality management marks a radical shift from traditional management to modern, data-driven leadership. This shift fosters transparency, adaptability, and continuous improvement, ultimately enhancing patient care and operational excellence. The detailed analyses of process mining, including organizational mining techniques, illuminate the complex interplay of resources, activities, and decisions within clinical workflows.

While our study focused on specific use cases, the potential applications of process mining in radiation oncology are vast. Future research could explore process mining for predictive modeling, risk assessment, and the development of personalized treatment plans. Additionally, integrating process mining with other data analytics tools and machine learning algorithms could unlock even more profound insights and optimize radiation oncology processes.

As the field of radiation oncology continues to evolve, process mining offers a robust framework for embracing data-driven decision-making, fostering innovation, and ultimately delivering high-quality and safe patient care. The transition from traditional management to modern leadership through process mining represents a crucial step toward a more efficient, effective, and patient-centric future for radiation oncology.

## Supporting information

S1 Appendix Key event log statistics. (DOCX)

S2 Appendix Detailed definitions of process modeling formalisms. (DOCX)

S3 Appendix Process mining algorithms. (DOCX)

S4 Appendix Conformance checking. (DOCX)
