# Supplementary material for: Utilizing process mining in quality management: A case study in radiation oncology
Source: PLOS Digit Health. 2025 May 15;4(5):e0000647. doi: 10.1371/journal.pdig.0000647 (PMC12080779; doi:10.1371/journal.pdig.0000647)
Supplement: S1 Appendix — (DOCX) [file pdig.0000647.s001.docx]

## Appendices

1. **Key Event Log Statistics**

Several statistics can be calculated from the event log to gain deeper insights into the process. These statistics can be grouped based on the primary requirements, additional resource attributes, and other contextual attributes [10].

### Statistics from the Three Main Requirements (Case ID, Activity, Timestamp)

- **Frequency of Activities**: Counts the number of times each activity occurs in the log.
- **Case Duration**: Measures the time to complete each case from start to end.
- **Throughput Time**: Measures the time taken from the start of the first activity to the end of the last activity for each case.
- **Activity Duration**: Measures the time taken for each activity to be completed.
- **Waiting Time**: Calculates the time spent waiting between consecutive activities in a case.
- **Trace Variants**: Identifies and counts unique sequences of activities (variants) in the log.
- **Start and End Activities**: Identifies the most common starting and ending activities in the log.
- **Activity Sequences**: Analyzes the sequences of activities and their frequencies.

### Statistics with the Resource Attribute

- **Resource Utilization**: Analyzes how often each resource is involved in activities.
- **Resource Workload**: Measures the number of activities each resource performs.
- **Resource Performance**: Evaluates the performance of resources based on the time taken to complete activities.
- **Handover of Work**: Analyzes the transitions between resources, identifying how work is passed from one resource to another.
- **Resource Availability**: Measures resource availability and idle times.
- **Resource Collaboration**: Identifies the collaboration patterns between resources, showing how often they work together on the same case.

### Statistics with Other Attributes (Technique, Modality, etc.)

- **Attribute-Based Performance**: Measures performance metrics (e.g., case duration, activity duration) based on different attributes like technique, age, and sex.
- **Attribute-Based Variants**: Analyzes how different attributes affect the variants in the event log.
- **Attribute Frequency**: Counts the occurrences of each attribute value in the log (e.g., number of cases for each technique, age group, sex).
- **Demographic Analysis**: Provides insights into how different demographic groups (age, sex) navigate the process.
- **Technique Efficiency**: Compares the efficiency of different techniques based on performance metrics like case duration and activity duration.
- **Correlation Analysis**: Identifies correlations between attributes (e.g., age, case duration, technique, and success rate).
- **Attribute-Based Deviations**: Analyzes deviations in the process based on different attributes, identifying which attributes are associated with higher deviation rates.
