# Supplementary material for: Utilizing process mining in quality management: A case study in radiation oncology
Source: PLOS Digit Health. 2025 May 15;4(5):e0000647. doi: 10.1371/journal.pdig.0000647 (PMC12080779; doi:10.1371/journal.pdig.0000647)
Supplement: S3 Appendix — (DOCX) [file pdig.0000647.s003.docx]

## Process Mining Algorithms [10]

### Alpha Miner

The Alpha Miner is a straightforward and well-liked process identification algorithm that creates a Petri net framework from a log of various events. It is designed to recover causation from a set of event sequences. The algorithm begins by determining each process instance’s beginning and ending activities. It then builds a Petri net model by repeatedly adding new transitions and places based on the behavior observed in the event log.

##### Steps of Alpha Miner

- - - 1. Identify the occurrence of each event.
      2. Define every potential start event.
      3. Define every potential end event.
      4. Calculate all possible sets of events that are independent of each other and causally connected.
      5. Eliminate every non-maximum set.
      6. Add start and finish places along with places for all derived sets.
      7. Add every arc.
      8. Return the Petri net.

### Directly Follows Graph

The Directly Follows Graph (DFG) graphically portrays the ordering relationships between events in the operation. It operates by building a directed graph that displays the ordering relationships and the frequency of each incident in the log. Although it does not offer a comprehensive process model, it provides a quick and practical approach to visualizing process behavior.

##### Explanation of Directly Follows Graph

- Each event in the event log (*L*) is represented as a node in the graph.
- Ordered edges connect the nodes, indicating the directly follows relationships (≻) between pairs of events (see Equation [1](#_bookmark0)).
- The weight of an edge represents the frequency of the directly following relationship between two nodes.

### Inductive Miner

Inductive Miner is a data-driven process discovery algorithm that uses machine learning methods to derive a workflow structure from the event log automatically. It establishes a methodology for recursively finding process trees in event logs. The algorithm builds a Petri net model that minimizes deviations from observed behavior and represents the tendencies of process instances in the event log.

##### Steps of Inductive Miner

- - - 1. Calculate the set of transitions with no incoming arcs, representing the initial marking in the Petri net.
      2. Identify the final markings by finding transitions with no outgoing arcs.
      3. Determine directly follows dependencies (≻) between pairs of events in the event log (see Equation [1](#_bookmark0)).
      4. Measure parallel dependencies (∥) between event pairs in the log (see Equation [2](#_bookmark1)).
      5. Calculate the set of choices between pairs of events in the log.
      6. Determine the number of causal connections among related events in the log, identifying direct contributions of events to the occurrence of subsequent events.

### Heuristic Miner

Heuristic Miner is a more complex process discovery algorithm that identifies the most plausible model from the event log using a mix of methods, including frequency analysis, causal relationships, and data clustering. It builds a Petri net model with the fewest possible model elements that accurately portray the characteristics of the process in the event log. The process is broken down into three stages: dependency graph mining, combining relations, and analyzing dependency interactions.

##### Steps of Heuristic Miner

- - - 1. **Frequency Analysis**: Determines the recurrence of events in the log.
      2. **Directly Follows Analysis**: Counts how often an event follows another event immediately in the log (≻) (see Equation [1](#_bookmark0)).
      3. **Parallelism Analysis**: Calculates how often two events co-occur without one directly following the other (∥) (see Equation [2](#_bookmark1)).
      4. **Choice Analysis**: Demonstrates how often one event follows another event in the log.
      5. **Loops Analysis**: Counts how often an event occurs twice in a row in the log.

## Conformance Checking [10]

Conformance checking assesses how well an event log aligns with a process model. It identifies discrepancies and measures the fitness, precision, and other conformance metrics. Below, we describe two key conformance checking techniques and explain the metrics used:

### Token-Based Replay

Token-based replay is a technique that replays the event log on the process model to identify deviations. Here’s a simplified step-by-step process of how token-based replay works:

1. Initialize Tokens: Place tokens in the initial place of the Petri net.
2. Replay Each Trace: For each trace *σ_i_* = ⟨*a*_1_*, a*_2_*, . . . , a_k_*⟩ in the event log: Move tokens through the transitions of the Petri net according to the sequence of activities in the trace. Consume tokens from the input places and produce tokens in the transitions’ output places.
3. Record Deviations: If a transition cannot fire because there are not enough tokens in its input places, record a missing token. If tokens are left in the places that should be empty by the end of the trace, record the remaining tokens.
4. Measure Fitness: Calculate the fitness measure based on the number of missing and remaining tokens. Fitness indicates how well the process model supports the event log.

This approach identifies where and how the actual process deviates from the modeled process, providing insights into process compliance and improvement areas.

### Alignments

Alignments provide a precise way to compare each trace in the event log with the process model by finding the optimal alignment between them. Here’s a simplified step-by-step process of how alignments work:

1. Construct the Alignment Matrix: For each trace *σ_i_* = ⟨*a*_1_*, a*_2_*, . . . , a_k_*⟩ in the event log and its corresponding sequence in the process model: Create an alignment matrix where rows represent the trace and columns represent the process model activities.
2. Define Costs: Define costs for different types of moves: Synchronous moves (matching moves): The trace and model execute the same activity. Log moves: The trace has an activity that the model does not. Model moves: The model has an activity that the trace does not.
3. Find Optimal Alignment: Use dynamic programming or other optimization techniques to find the alignment with the minimum total cost, balancing synchronous, log, and model moves.
4. Measure Conformance: Calculate conformance metrics based on the alignment.
