# Supplementary material for: Utilizing process mining in quality management: A case study in radiation oncology
Source: PLOS Digit Health. 2025 May 15;4(5):e0000647. doi: 10.1371/journal.pdig.0000647 (PMC12080779; doi:10.1371/journal.pdig.0000647)
Supplement: S4 Appendix — (DOCX) [file pdig.0000647.s004.docx]

## Conformance Checking [10]

Conformance checking assesses how well an event log aligns with a process model. It identifies discrepancies and measures the fitness, precision, and other conformance metrics. Below, we describe two key conformance checking techniques and explain the metrics used:

### Token-Based Replay

Token-based replay is a technique that replays the event log on the process model to identify deviations. Here’s a simplified step-by-step process of how token-based replay works:

1. Initialize Tokens: Place tokens in the initial place of the Petri net.
2. Replay Each Trace: For each trace *σ_i_* = ⟨*a*_1_*, a*_2_*, . . . , a_k_*⟩ in the event log: Move tokens through the transitions of the Petri net according to the sequence of activities in the trace. Consume tokens from the input places and produce tokens in the transitions’ output places.
3. Record Deviations: If a transition cannot fire because there are not enough tokens in its input places, record a missing token. If tokens are left in the places that should be empty by the end of the trace, record the remaining tokens.
4. Measure Fitness: Calculate the fitness measure based on the number of missing and remaining tokens. Fitness indicates how well the process model supports the event log.

This approach identifies where and how the actual process deviates from the modeled process, providing insights into process compliance and improvement areas.

### Alignments

Alignments provide a precise way to compare each trace in the event log with the process model by finding the optimal alignment between them. Here’s a simplified step-by-step process of how alignments work:

1. Construct the Alignment Matrix: For each trace *σ_i_* = ⟨*a*_1_*, a*_2_*, . . . , a_k_*⟩ in the event log and its corresponding sequence in the process model: Create an alignment matrix where rows represent the trace and columns represent the process model activities.
2. Define Costs: Define costs for different types of moves: Synchronous moves (matching moves): The trace and model execute the same activity. Log moves: The trace has an activity that the model does not. Model moves: The model has an activity that the trace does not.
3. Find Optimal Alignment: Use dynamic programming or other optimization techniques to find the alignment with the minimum total cost, balancing synchronous, log, and model moves.
4. Measure Conformance: Calculate conformance metrics based on the alignment.
